# Comment on “Dobutamine-assisted exclusion of atrial appendage thrombus in atrial fibrillation patients with sludge”

**DOI:** 10.1016/j.ahjo.2026.100959

**Published:** 2026-10-14

**Authors:** R. Jayanthi, P. Bharathi, A. Chindoja, M. Regi Cindrella

**Affiliations:** a Meenakshi Medical College Hospital & Research Institute, Meenakshi Academy of Higher Education and Research, Enathur, Kanchipuram, Tamil Nadu, 631552, India; b Meenakshi College of Nursing, Meenakshi Academy of Higher Education and Research, Enathur, Kanchipuram, Tamil Nadu, India

**Keywords:** Atrial fibrillation, Left atrial appendage, Atrial appendage thrombus, Sludge, Dobutamine, Transesophageal echocardiography, Thrombus detection, Cardioversion, Ablation, Cardiac imaging

Dear Editor

Minami et al. [1], Gave dobutamine during echocardiography to 20 patients with atrial fibrillation and left atrial appendage sludge and report that it reduced sludge and facilitated exclusion of thrombus without serious adverse events. Sludge that hides the appendage is an obstacle before cardioversion, ablation or closure and a prospective registered protocol is welcome. The conclusions however go further than a single-arm study without a reference standard can support.

The study cannot show that dobutamine improves thrombus detection because no thrombus was found in any patient and no independent method confirmed their absence [2]. A technique that never identifies a thrombus provides no estimate of sensitivity. Clearer images after infusion show that the appendage became easier to see not that a thrombus would have been seen if present. Confirmation by contrast echocardiography computed tomography or direct inspection during closure which five patients underwent would give the finding a reference point.

How images were graded also matters. The first observer graded echo contrast during the procedure and therefore knew the timing of each image [3]. The second a co-author reviewed stored images blinded to data but baseline and post-infusion loops are easy to distinguish. Gain was adjusted to minimize noise. If it changed between acquisitions the apparent fall in echo density could partly reflect settings rather than flow. Stating whether gain was fixed would help.

The safety conclusion rests on 20 patients. With no events the upper bound of the 95% confidence interval for a complication is roughly 15% [4]. Than half the patients had a history of stroke or thromboembolism increased contractility in a sludge-filled appendage could in principle dislodge material and brain imaging was not performed as the authors acknowledge. The absence of events is reassuring but does not establish safety.

The explanation offered for the contrast with earlier reports in which sludge persisted despite dobutamine sits uneasily with the cohort described. The authors suggest their patients had advanced atrial cardiomyopathy. Yet 65% had standing persistent atrial fibrillation median left atrial reservoir strain was 7.0% and emptying fraction 19.0% and 80% had a history of heart failure. These content describe atrial disease so the uniform response remains unexplained and may reflect selection. Only 20 of 1142 patients undergoing the examination were. The text does not say how many others with sludge were screened out.

Some information should be reconciled. Baseline left ventricular ejection fraction is given as 51.8% in the Abstract and 51.6% in the Results and age and heart rate are described as means in one place and medians in another with an age range of 67 to 82 that matches the range.

These points bear on practice. If dobutamine is adopted to clear the way for procedures the key question is whether it can reveal thrombi reliably and safely not whether it improves image quality. A study including patients, with confirmed thrombi and an independent reference standard would answer that.

## CRediT authorship contribution statement

**R. Jayanthi:** Data curation. **P. Bharathi:** Conceptualization. **A. Chindoja:** Validation. **M. Regi Cindrella:** Formal analysis.

## Ethical approval

Not applicable (commentary on previously published research).

## Declaration of Generative AI and AI-assisted technologies in the writing process

Generative AI tools were used solely for language refinement and formatting assistance. All scientific interpretation and critique were independently developed by the authors.

## Funding

None.

## Declaration of competing interest

The authors declare no conflicts of interest.

## Data Availability

No new data were generated or analysed in this work.
